# Recombinant BCG-LTAK63 Vaccine Candidate for Tuberculosis Induces an Inflammatory Profile in Human Macrophages

**DOI:** 10.3390/vaccines10060831

**Published:** 2022-05-24

**Authors:** Carina C. dos Santos, Kimberley V. Walburg, Suzanne van Veen, Louis G. Wilson, Carlos E. M. Trufen, Ivan P. Nascimento, Tom H. M. Ottenhoff, Luciana C. C. Leite, Mariëlle C. Haks

**Affiliations:** 1 Laboratório de Desenvolvimento de Vacinas, Instituto Butantan, São Paulo 05503-900, Brazil; ivanpnbutantan@gmail.com; 2 Programa de Pós-Graduação Interunidades em Biotecnologia, Universidade de São Paulo, São Paulo 05508-900, Brazil; 3 Department of Infectious Diseases, Leiden University Medical Center, 2333 ZA Leiden, The Netherlands; k.v.walburg@lumc.nl (K.V.W.); s.van_veen@lumc.nl (S.v.V.); glwilson03@gmail.com (L.G.W.); t.h.m.ottenhoff@lumc.nl (T.H.M.O.); mchaks@lumc.nl (M.C.H.); 4 Department of Clinical and Toxicological Analysis, Faculty of Pharmacy, Federal University of Bahia, Salvador 40170-115, Brazil; 5 Czech Centre for Phenogenomics, 25250 Vestec, Czech Republic; trufenc@img.cas.cz

**Keywords:** tuberculosis, BCG vaccination, recombinant BCG, gene expression profiling, cytokine profiling, primary human macrophages, immune response

## Abstract

Tuberculosis (TB) is one of the top 10 leading causes of death worldwide. The recombinant BCG strain expressing the genetically detoxified A subunit of the thermolabile toxin from *Escherichia coli* (LTAK63) adjuvant (rBCG-LTAK63) has previously been shown to confer superior protection and immunogenicity compared to BCG in a murine TB infection model. To further investigate the immunological mechanisms induced by rBCG-LTAK63, we evaluated the immune responses induced by rBCG-LTAK63, BCG, and *Mycobacterium tuberculosis* (*Mtb*) H37Rv strains in experimental infections of primary human M1 and M2 macrophages at the transcriptomic and cytokine secretion levels. The rBCG-LTAK63-infected M1 macrophages more profoundly upregulated interferon-inducible genes such as *IFIT3*, *OAS3*, and antimicrobial gene *CXCL9* compared to BCG, and induced higher levels of inflammatory cytokines such as IL-12(p70), TNF-β, and IL-15. The rBCG-LTAK63-infected M2 macrophages more extensively upregulated transcripts of inflammation-related genes, *TAP1*, *GBP1*, *SLAMF7*, *TNIP1*, and *IL6*, and induced higher levels of cytokines related to inflammation and tissue repair, MCP-3 and EGF, as compared to BCG. Thus, our data revealed an important signature of immune responses induced in human macrophages by rBCG-LTAK63 associated with increased inflammation, activation, and tissue repair, which may be correlated with a protective immune response against TB.

## 1. Introduction

Tuberculosis (TB) is still a public health problem and is one of the top 10 causes of death in the world. The COVID-19 pandemic has reversed years of progress in providing essential TB services and reducing TB disease burden. There was a large global drop in the number of people newly diagnosed with TB and reported in 2020 as compared with 2019; this fell from 7.1 million in 2019 to 5.8 million in 2020. Consequently, infected and non-diagnosed patients did not receive treatment, resulting in an increase in the number of people who died from TB in 2020. There were an estimated 1.3 million deaths among HIV-negative people and an additional 214,000 deaths among HIV-positive people [1]. The BCG vaccine does not sufficiently prevent pulmonary tuberculosis in adults [2]; thus, new prevention or treatment strategies are required to control the infection spread, and these are part of the main goals of the World Health Organization’s End TB Strategy [1].

There are several factors that contribute to the success of *Mycobacterium tuberculosis* (*Mtb*) as the main cause of TB. One of them is that this pathogen employs several mechanisms to evade induced immune responses [3]. Classically, it is known that *Mtb* primarily infects macrophages and can inhibit phagosome maturation by arresting phagosome–lysosome fusion to ensure intracellular survival within these innate immune cells [4]. Furthermore, it has been reported that *Mtb* can modulate the microenvironment of the initial immune response, reprogram macrophages, and delay antigen presentation [5]. Macrophages are heterogeneous cells that have originally been subdivided into classically activated M1 macrophages and alternative activated M2 macrophages as being the polar ends of a complex polarization spectrum. They have been described to differ in terms of receptors, cytokine and chemokine expression, and effector functions. M1 macrophages are mainly involved in microbicidal and inflammatory mechanisms, while M2 macrophages are predominantly engaged in immunomodulatory and tissue homeostatic activities. However, macrophages are highly plastic innate immune cells, and depending on available environmental stimuli, M1 macrophages can redifferentiate into M2 cells or vice versa [6,7,8,9].

The innate immune response is important for the development of an effective adaptive immune response, which can display immunological memory. Moreover, in recent years, studies on innate immunity and patterns of macrophage activation have gained more attention, and they play an important role in the development of new vaccine strategies against TB [10]. The rBCG-LTAK63 strain, one of the next-generation TB vaccine candidates based on Bacillus Calmette–Guérin (BCG) [11], was previously shown to confer enhanced protection against an intratracheal challenge with *Mtb* in mice as compared to BCG [12]. The rBCG-LTAK63 strain takes advantage of the adjuvant properties of LTAK63. The heat-labile *Escherichia coli* enterotoxin and its derivatives have a broad spectrum of adjuvant properties, which can improve the innate and adaptive immune responses [13,14]. These results indicate the potential of rBCG-LTAK63 as a vaccine candidate. In order to move forward towards clinical applications, we have recently constructed an unmarked strain of rBCG expressing LTAK63 through auxotrophic complementation. The BCG auxotroph, obtained by the CRISPR/cas9 approach, was transformed with a complementation vector expressing the antigen without an antibiotic resistance marker [15]. This strain will be suitable for clinical studies.

Additional investigation showed that the immunization of mice with the rBCG-LTAK63 strain increased the recruitment of neutrophils, macrophages, and lymphocytes, and induced enhanced innate and long-term adaptive immune responses. In addition, increased levels of mediators and effectors of macrophage activation, such as nitric oxide, hydrogen peroxide, and inflammatory cytokines, were observed in rBCG-LTAK63-immunized animals [16].

To further investigate the immune mechanisms induced by rBCG-LTAK63, and reinforce its potential as a human vaccine, we explored and compared the immune responses induced by rBCG-LTAK63, BCG, and *Mtb* H37Rv strains in an in vitro infection model of primary human M1 and M2 macrophages. Macrophage responses in both cell subtypes were analyzed based on gene expression and cytokine secretion profiles.

## 2. Materials and Methods

### 2.1. Culture of Primary Human-Monocyte-Derived Macrophages

Buffy coats were obtained from healthy donors after written informed consent (Sanquin Blood Bank, Amsterdam, The Netherlands). Monocytes were isolated from buffy coats by FICOLL separation and CD14 MACS sorting (Miltenyi Biotec, Teterow, Germany). The isolated CD14+ cells were subsequently cultured in Gibco Roswell Park Memorial Institute (RPMI) 1640 medium (Gibco, Life Technologies, Paisley, UK) containing 10% FBS, 2 mM L-alanyl-L-glutamine (PAA, Linz, Austria), and either 5 ng/mL granulocyte–macrophage-colony stimulating factor (GM-CSF, BioSource Life Technologies-Invitrogen, Waltham, MA, USA) to generate M1 macrophages or 50 ng/mL macrophage-colony stimulating factor (M-CSF, R&D Systems, Abingdon, UK), to generate M2 macrophages [17,18]. Following differentiation for 6 days at 37 °C/5% CO_2_, macrophages were harvested using Trypsin (Sigma-Aldrich^®^, Merck KGaA, St. Louis, MO, USA) and scrapping and analyzed by flow cytometry for the expression of cell surface markers CD14, CD11b, and CD163.

### 2.2. Bacterial Culture and Macrophage Infection

All experimental procedures were performed according to local and national guidelines for working with pathogenic mycobacteria. *Mycobacterium bovis* BCG Moreau strain was used to generate the recombinant rBCG-LTAK63 strain, as previously described [12]. BCG wild-type, rBCG-LTAK63, and *Mtb* H37Rv strains were grown in Middlebrook 7H9 medium (MB7H9) (Difco, Detroit, MI, USA) supplemented with albumin–dextrose–catalase broth (ADC) (BBL, Cockeysville, MD, USA) and 0.05% Tween 80 (Sigma Chemical Co., St. Louis, MO, USA) (MB7H9/ADC/Tw) and 20 µg/mL kanamycin (for rBCG-LTAK63). Cultures were incubated at 37 °C with 5% CO_2_ until they reached an optical density of 1.0 at 600 nm (OD600).

One day before infection, M1 and M2 macrophages were plated in either 48= or 96-well plates at a density of 1 × 10^5^ cells/well. In addition, mycobacterial cultures were diluted to a density corresponding to early log-phase growth OD600 of 0.4. On the day of infection, M1 and M2 macrophage cultures were inoculated with 100 μL of rBCG-LTAK63, BCG, or *Mtb* H37Rv (multiplicity of infection (MOI) = 10) and incubated at 37 °C with 5% CO_2_ for 1, 24, or 48 h, as indicated.

### 2.3. Intracellular Mycobacterial Survival Assay

Macrophages were infected with mycobacterial strains for 1, 24, or 48 h. At each time point, the supernatant was removed, and cells were washed with RPMI containing a high gentamicin concentration (50 µg/mL), followed by incubation for 2 h with RPMI containing a low gentamicin concentration (5 µg/mL) to eliminate extracellular bacteria. Then, the supernatant was removed, and cells were lysed using H_2_O containing 0.05% SDS. Serial dilutions were plated on Middlebrook 7H10 medium (MB7H10) (Difco, Detroit, MI, USA) supplemented with oleic–albumin–dextrose–catalase broth (OADC) (BBL, Cockeysville, MD, USA) and 20 µg/mL kanamycin for rBCG-LTAK63. After 14 days, colony-forming units (CFUs) were counted.

### 2.4. RNA Extraction, dcRT-MLPA Assay, and Data Analysis

Macrophages were infected with mycobacterial strains for 24 or 48 h. At each time point, infected cells were collected in 350 µL of Trizol (Life Technologies-Invitrogen), and the RNA was isolated according to the manufacturer’s protocol. Briefly, cells were incubated for 5 min at room temperature (RT). Chloroform (70 µL) was added, and the mixture was centrifuged for 15 min at 12,000× *g* at 4 °C. The aqueous phase was transferred to a 1.5 mL microcentrifuge tube. Isopropanol (175 µL) was added and incubated for 20 min at RT. The samples were spun for 10 min at 12,000× *g* at 4 °C, and the RNA pellets were washed twice with 1 mL of ethanol 75% before being dissolved in RNase-free H_2_O (12 µL). The concentration of RNA was measured using a Nanodrop, and the RNA was stored at −80 °C until downstream processing.

Dual-color reverse transcriptase multiplex ligation-dependent probe amplification (dcRT-MLPA) was performed as described previously [19,20]. Briefly, for each target-specific sequence, a specific RT primer was designed to hybridize immediately downstream of the left- and right-hand half-probe target sequence. Following reverse transcription of 100 ng RNA (M1 macrophages) or 25 ng RNA (M2 macrophages) using MMLV reverse transcriptase (Promega, Leiden, the Netherlands), left- and right-hand half-probes were hybridized to the cDNA at 60 °C overnight. Annealed half-probes were ligated and subsequently amplified by PCR (33 cycles of 30 s at 95 °C, 30 s at 58 °C, and 60 s at 72 °C, followed by 1 cycle of 20 min at 72 °C). Primers and probes used were from Sigma-Aldrich and MLPA reagents from MRC Holland (Amsterdam, the Netherlands). PCR amplification products were diluted 1:10 in HiDi formamide (Thermo Fisher Scientific, Waltham, MA, USA) containing GeneScan 400HD ROX (Applied Biosystems, Waltham, MA, USA) size standard and were analyzed on an Applied Biosystems 3730 capillary sequencer in GeneScan mode (Baseclear, Leiden, the Netherlands). Trace data were analyzed using GeneMapper software package 5.0 (Applied Biosystems/Life Technologies). The areas of each assigned peak (in arbitrary units) were exported for further analysis in Microsoft Excel spreadsheet software. Signals below the threshold value for noise cutoff in GeneMapper (log2-transformed peak area ≤7.64) were assigned to the threshold value for noise cutoff. Following normalization of the data to the average signal of housekeeping gene GAPDH, signals below the threshold value for noise cutoff in GeneMapper were again assigned the threshold value for noise cutoff. Primers and probes of 43 preselected immune-associated genes were designed by Leiden University Medical Center (LUMC). The fold change was calculated by comparing infected and uninfected macrophages for all genes, and statistical analysis was performed as indicated.

### 2.5. Cytokine Production

Macrophages were infected with mycobacterial strains, and the supernatant was collected 48 h after infection. Production of the following cytokines were measured using the multiplex cytokine assay, Milliplex (Merck Millipore, Burlington, MA, USA) according to the manufacturer’s protocol: IL12(p70), TGF-α, MCP-3, MDC, TNF-β, Eotaxin, Fractalkine, SCD40L, IL-1α, IL-2, IL-4, IL-3, IL-5, IL-9, IL-10, IL-15, IL-17, IFN-γ, IP-10, TNF-α, IL-1β, IL-6, IL12(p40), IL-7, IL-13, IFNA2, TGF-α, EGF, VEGF, FGF-2, PDGF-AA, PDGF-AB-BB, FLT-3L, G-CSF, GM-CSF, RANTES.

### 2.6. Statistical Analysis

Statistical significance was determined by paired Student’s *t*-test or repeated-measures (RM)-ANOVA with *p* ≤ 0.05 or indicated *p*-value, as described in the figure captions. GraphPad Prism (version 7.02, Prism, La Jolla, CA, USA) was used for statistical analysis.

## 3. Results

### 3.1. Intracellular Survival of rBCG-LTAK63 in M1 and M2 Macrophages Is Comparable to BCG

To investigate the survival of the rBCG-LTAK63 strain in M1 and M2 macrophages in comparison with BCG, intracellular mycobacteria were recovered at different time points post-infection and counted by CFU. The CFU recovered from M1 and M2 macrophages infected with rBCG-LTAK63 was comparable to those infected with BCG (Supplementary Figure S1A), indicating similar survival and growth rates. Furthermore, the expression of the vector containing the LTAK63 gene was stable in both macrophage subsets over 48 h (the latest time point in our human in vitro infection model), as demonstrated by similar CFU counts of rBCG-LTAK63 in the presence or absence of kanamycin (Supplementary Figure S1B).

### 3.2. M1 Macrophages Infected with rBCG-LTAK63 Display Marked Upregulation of Genes Associated with the IFN Signaling Pathway

**Figure 1:**
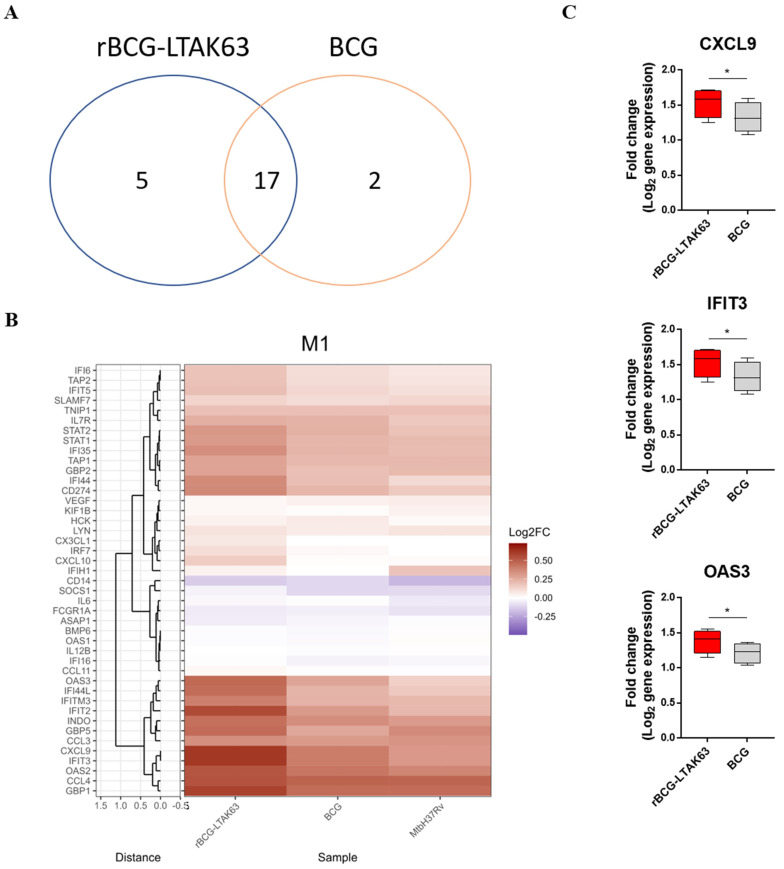
M1 macrophages infected with rBCG-LTAK63 display marked upregulation of genes associated with IFN signaling pathway. M1 macrophages from 4 different donors were infected with rBCG-LTAK63, BCG, or *Mtb* H37Rv, and transcriptomic profiles were determined at 24 h post-infection by dcRT-MLPA. (**A**) Venn diagram displaying the number of differentially expressed genes (*p* ˂ 0.05) identified in rBCG-LTAK63- or BCG-infected M1 macrophages (fold change in relation to uninfected M1 macrophages). (**B**) Heat map showing fold changes in expression profile of the 43 immune-related genes in M1 macrophages in response to infection with rBCG-LTAK63, BCG, or *Mtb* H37Rv. (**C**) Differentially expressed genes (*p* ˂ 0.05) in rBCG-LTAK63-infected M1 macrophages when compared to BCG-infected M1 cells. Statistical significance was determined by paired Student’s *t*-test. Significant differences were observed as indicated * *p* ˂ 0.05.

The gene expression profiles of 43 immune-associated genes were evaluated by dcRT-MLPA in macrophages infected with BCG, rBCG-LTAK63, or *Mtb* H37Rv (Supplementary Figure S2, Figure 1B). At 24 h post-infection, 22 DEGs were identified in M1 macrophages infected with rBCG-LTAK63 (compared to uninfected controls), while 19 DEGs were identified in BCG-infected M1 cells, and 17 of these DEGs were found to be in common (Figure 1A). Interestingly, a large set of interferon (IFN)-signaling genes was more profoundly induced in rBCG-LTAK63-infected M1 as compared to either BCG- and *Mtb*-infected M1 (Figure 1B, Supplementary Table S1). The IFIT3 and OAS3 genes were significantly upregulated when rBCG-LTAK63 was compared to BCG-infected M1. CXCL9, an antimicrobial gene, was also significantly upregulated in rBCG-LTAK63-infected M1 as compared to BCG-infected M1 macrophages (Figure 1C).

### 3.3. BCG Induces Downregulation of an Inflammatory Gene Profile in M2 Macrophages, While rBCG-LTAK63 Maintains or Induces Upregulation for 48 h

**Figure 2:**
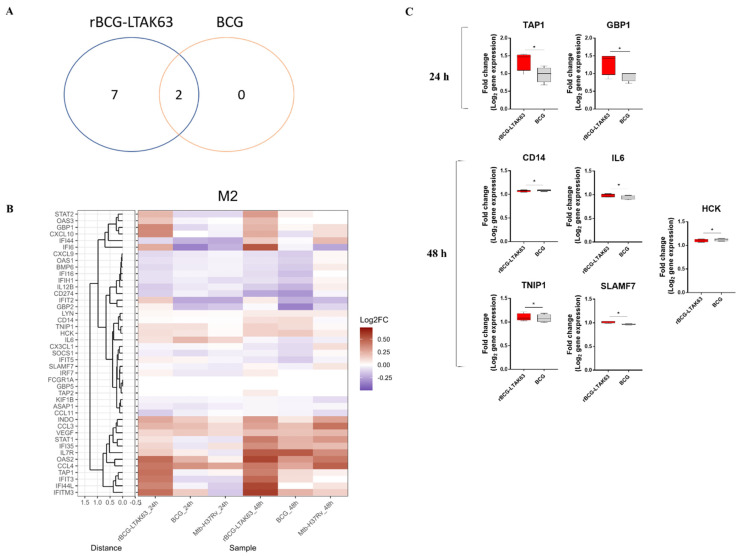
BCG infection downregulates an inflammatory gene profile in M2 macrophages 48 h post-infection, while rBCG-LTAK63 infection maintains or further upregulates genes associated with inflammation. M2 macrophages derived from 4 different donors were infected with rBCG-LTAK63, BCG, or *Mtb* H37Rv, and transcriptomic profiles were determined at 24 h and 48 h post-infection by dcRT-MLPA. (**A**) Venn diagram displaying the number of differentially expressed genes (*p* ˂ 0.05) identified in rBCG-LTAK63- or BCG-infected M2 macrophages at 48 h (fold change in relation to uninfected M2 macrophages). (**B**) Heat map showing fold changes in expression profile of the 43 immune-related genes in M2 macrophages in response to infection with rBCG-LTAK63, BCG, or *Mtb* H37Rv for 24 h or 48 h. (**C**) Differentially expressed genes (*p* ˂ 0.05) in rBCG-LTAK63-infected M2 macrophages compared to BCG-infected M2 macrophages. Statistical significance was determined by paired Student *t*-test. Significant differences were observed as indicated * *p* ˂ 0.05.

In contrast to M1 macrophages, only very few DEGs could be identified in mycobacteria-infected M2 macrophages at 24 h post-infection compared to uninfected controls (Supplementary Table S2, Figure 2B). Nevertheless, similar to M1, IFN-signaling genes were more profoundly induced in rBCG-LTAK63-infected M2 compared to either BCG- or *Mtb*-infected M2. Moreover, the IFN-signaling TAP1 and GBP1 genes were found to be significantly upregulated in rBCG-LTAK63-infected M2 when compared to BCG-infected M2 macrophages (Figure 2B,C). After 48 h, 9 DEGs could be identified in M2 macrophages infected with rBCG-LTAK63 (compared to uninfected controls), while 2 DEGs could be identified in M2 macrophages infected with BCG, which were DEGs in common with rBCG-LTAK63-infected M2 (Figure 2A). Of note, at 48 h post-infection, BCG-infected M2 macrophages had predominantly downregulated inflammatory-correlated genes, while rBCG-LTAK63 infection maintained or further upregulated inflammatory-associated transcripts. Expression of the genes, TNIP1, SLAMF7, and IL6 was upregulated and CD14 and HCK genes downregulated when rBCG-LTAK63 was compared to BCG-infected M2 macrophages (Figure 2C).

### 3.4. M1 and M2 Macrophages Infected with rBCG-LTAK63 Display Increased Production of Inflammatory Cytokines

Cytokines are mediators and effector molecules of immune responses against pathogens. We evaluated the production of cytokines associated with inflammatory responses, cell growth, and repair in the culture supernatants of M1 and M2 macrophages infected with rBCG-LTAK63 or BCG at 48 h post-infection, or uninfected macrophages as a control.

**Figure 3:**
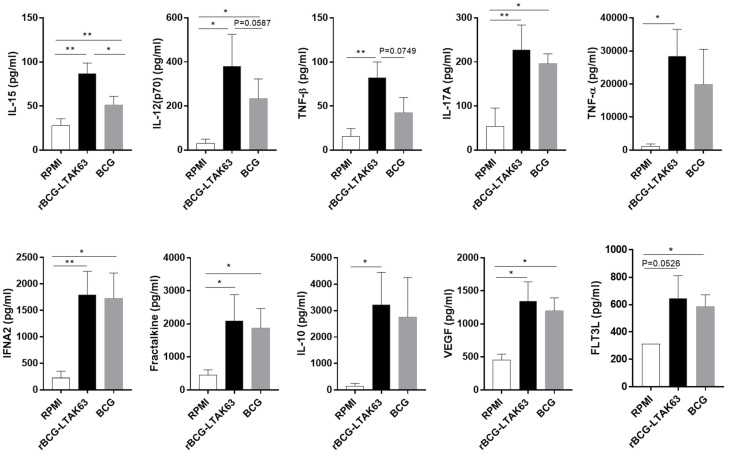
M1 macrophages infected with rBCG-LTAK63 display higher secretion levels of inflammatory cytokines. M1 macrophages derived from 4 different donors were infected with rBCG-LTAK63 or BCG for 48 h and the supernatants were analyzed for cytokine production by Luminex and compared to uninfected control cells. Statistical significance was determined by RM—ANOVA (*p* ˂ 0.05). Significant differences were observed as indicated * *p* ˂ 0.05, ** *p* ˂ 0.01.

Overall, the levels of secreted cytokines in rBCG-LTAK63-infected M1 macrophages clearly surpassed the levels observed in BCG-infected M1. Furthermore, the production of IL-17A, IFNA2, TNF-α, Fractalkine, IL-10, VEGF, and FLT3L was strongly induced in both rBCG-LTAK63- and BCG-infected M1 when compared with uninfected M1 macrophages but, more interestingly, the production of IL-15 and proinflammatory cytokines IL-12(p70) and TNF-β was significantly higher in rBCG-LTAK63- compared to BCG-infected M1 macrophages (Figure 3).

**Figure 4:**
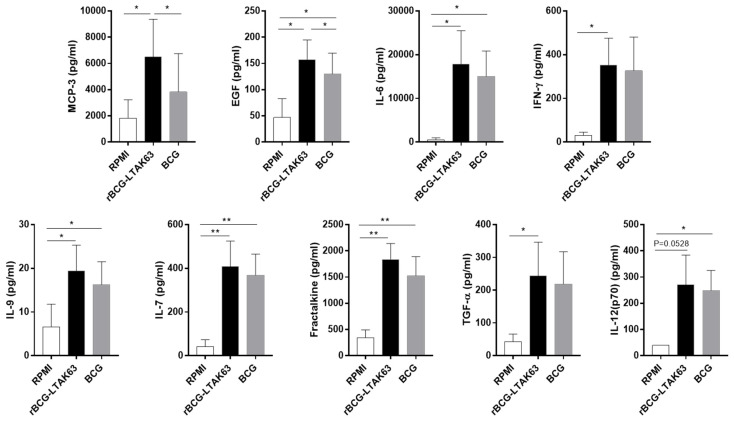
M2 macrophages infected with rBCG-LTAK63 display higher secretion levels of inflammatory cytokines. M2 macrophages derived from 4 different donors were infected with rBCG-LTAK63 or BCG for 48 h and the supernatants were analyzed for cytokine production by Luminex and compared to uninfected controls. Statistical significance was determined by RM—ANOVA (*p* ˂ 0.05). Significant differences were observed as indicated * *p* ˂ 0.05, ** *p* ˂ 0.01.

Similar to M1-infected cells, overall, the levels of secreted cytokines in rBCG-LTAK63-infected M2 macrophages exceeded the levels observed in BCG-infected M2 (Figure 4). The production of MCP-3 and EGF was higher in rBCG-LTAK63 when compared with BCG-infected M2 macrophages; the production of IFN-γ was higher only when rBCG-LTAK63 was compared with uninfected M2 macrophages; and the production of IL-6, IL-9, IL-7, Fractalkine, TGF-α, and IL-12 (p70) was higher in rBCG-LTAK63 or BCG when compared with uninfected M2 macrophages.

## 4. Discussion

In order to uncover the immunological mechanisms or pathways activated by rBCG-LTAK63 in human cells and in more depth, we investigated the gene expression profiles induced in infected primary human macrophages. Since the expression of heterologous proteins in bacteria can be influenced by several factors in both in vivo and in vitro conditions [21,22,23], we first demonstrated that the recombinant strain is stable during infection of primary human macrophages.

Compared to BCG-infected M1 cells, the rBCG-LTAK63-infected M1 macrophages displayed stronger upregulation of IFIT3 and OAS3 genes, which are part of the IFN signaling pathway. The role of the IFN signaling pathway is complex, as it can both be beneficial or harmful to the host, depending on the experimental context [24]. The *IFIT3* gene is part of the interferon-induced proteins with tetratricopeptide repeats (IFITs) family, strongly induced due to activation of type I IFN [25]. Initially, the role of this gene was described in antiviral defense, as well as in the activation of several components of the IFN signaling pathway [24]. However, it has also been described to be positively regulated in bacterial infections, such as mycobacteria infections [26,27,28,29].

Expression of IFIT family genes depends on pattern recognition and the JAK-STAT pathway. Pathogen-associated molecular patterns (PAMPs) are molecules associated with different groups of pathogens, including viruses, bacteria, fungi, and others. Pattern recognition receptors (PRRs) recognize different PAMPs during infection by pathogens and activate signaling molecules. As a result, activation of receptors known as Toll-like (TLRs) and RIG-like receptors (RLRs) induce gene expression of the IFIT family [25]. The IFIT3 gene has been described as a protective molecule against virus infection [27]. It has been reported that upon viral or bacterial infection, the IFN signaling activation induces the production of the 2′-5′-oligoadenylate synthetase (OAS) family [30], which includes the *OAS3* gene, that was upregulated by infection with rBCG-LTAK63. The role of the OAS family in bacterial infections is not well defined yet. However, it has been reported to induce intracellular survival of mycobacteria, IFN secretion, and autophagy [31].

Another gene significantly upregulated by infection with rBCG-LTAK63 compared to BCG is the IFN-induced monokine-encoding gene, *CXCL9*, that is induced in response to IFN-γ and induces inflammation together with recruitment of activated lymphocytes [32,33]. CXCL9 is a pleiotropic molecule involved in innate and adaptive immune response mechanisms [34,35,36].

The higher protein secretion levels of IL-12(p70), TNF-β, and IL-15 displayed in rBCG-LTAK63-infected M1 are in agreement with the findings at the transcriptomic level in that infection with rBCG-LTAK63 enhanced the inflammatory and activated cellular immune response profile compared to BCG-infected M1 macrophages. IL-12(p70) is a proinflammatory cytokine that has been described to have an important role in the control of mycobacterial infections [37,38,39]. The proinflammatory cytokines, TNF-β and TNF-α, are homotrimers, and they have similar structures and functions [40,41]. It has also been reported that mycobacteria-infected macrophages secrete IL-15 [42]. It is mainly produced by macrophages and dendritic cells and acts in the recruitment of T lymphocytes, stimulating CD4 T cells [43] and specific subpopulations of memory lymphocytes, such as CD8 T cells [44,45]. In support of a possible role for IL-15 during rBCG-LTAK63 vaccination, we have previously shown that rBCG-LTAK63 induces CD4+ and CD8+ T cells in vivo [16]. However, the induction of memory and activation markers for T cells still need to be evaluated.

It is interesting to note the unique feature of the rBCG-LTAK63 induced transcriptomic signature, which separates it from BCG and *Mtb*, and which involves the strong induction of a large set of IFN signaling genes. Although excessive inflammation may be damaging to the host [46], it is evident that neither BCG nor *Mtb* induces an immune response that effectively eliminates *Mtb.* On the other hand, our previous results have shown that immunization with rBCG-LTAK63 induced reduced immunopathology in *Mtb*-infected mouse lungs [16]. Therefore, our results suggest that the superior induction of IFN signaling genes/proinflammatory cytokines by rBCG-LTAK63 correlates with, and may at least partly explain, the improved protection in mice.

Since M2 macrophages usually have an immunomodulatory phenotype, a different gene signature and cytokine production profile would be expected. While genes associated with an inflammatory profile were rapidly upregulated in mycobacteria-infected M1 macrophages (within 24 h), mycobacteria-infected M2 macrophages showed only few DEGs 24 h post-infection. Substantially more DEGs were identified at 48 h post-infection, suggesting that the default immunomodulatory profile of M2 macrophages delays the switch towards an inflammatory profile.

In M2 macrophages, genes associated with an inflammatory profile were more extensively upregulated by infection with rBCG-LTAK63, compared to infection with BCG, including the *TAP1* and *GBP1* genes, which are both part of the IFN signaling pathway. TAP1—transporter associated with antigen processing-1—is important for MHC-I function and has a key role in adaptive immunity [47,48]. GBP1—guanylate-binding protein-1 precursor—is part of the IFN-induced GTPase family. This protein is associated with protection against bacterial infections, host defense, and intracellular pathogen killing, including mycobacteria [49,50,51,52].

The levels of expression of the *TNIP1, SLAMF7*, and *IL6* genes were also higher in M2 infected with rBCG-LTAK63 when compared to BCG. TNIP1 is one of the major regulators of the NF-Κβ signaling pathway and is implicated in cell inflammation [53]. A wide repertoire of functions is suggested for TNIP1, including modulation of cellular activation and enhancement of CD4 T-cell levels [54]. IL-6 is a pleiotropic cytokine, secreted by a variety of cell types, playing a role in inflammation, response to infections, and repair of cellular tissues [55]. SLAMF7 is a receptor present in NK cells and is related to cell activation [56,57]. Furthermore, the cytokine MCP-3 was produced at elevated levels in M2 macrophages infected with rBGC-LTAK63. The chemokine MCP-3 is structurally and functionally similar to MCP-1, and both are potent monocyte chemoattractants for T cells and NK cells [58]. On the whole, these results could suggest that rBCG-LTAK63 activated M2 cells may have a role in signaling to NK and CD4 T cells; however, the involvement of NK cells has not been investigated. The cytokine EGF was produced at elevated levels in M2 macrophages infected with rBGC-LTAK63 as compared to those infected with BCG. Epidermal growth factor (EGF) promotes proliferation, differentiation, survival, and repair in several cell types [59].

**Figure 5:**
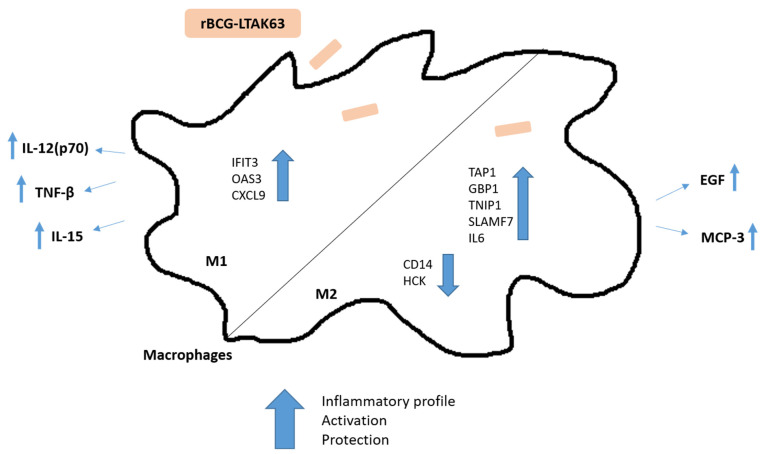
Schematic representation of the immune responses induced in human macrophages by infection with rBCG-LTAK63 that could correlate with the superior protection observed in a murine TB challenge model. Displayed are the differentially expressed genes (IFIT3, OAS3, CXCL9, TAP1, GBP1, TNIP1, SLAMF7, IL6, CD14, and HCK) and the production of cytokines (IL-12(p70), TNF-β, IL-15, MCP-3, and EGF).

In conclusion, rBCG-LTAK63, when compared to BCG, induced a dominant inflammatory profile in M1 macrophages, while a combined inflammatory and repair profile was induced in M2 macrophages. In this study, we used three different mycobacterial strains: *Mtb* H37Rv, a virulent strain; BCG, the current attenuated strain used as a vaccine for TB; and the rBCG-LTAK63, a recombinant BCG strain that has been shown to confer increased protective properties against TB in a murine TB challenge model. In general, we observed only few differences between the attenuated BCG and the virulent *Mtb* strains, but this is in agreement with other studies that have shown that infection, even with inactivated pathogens, is able to activate immune responses [60]. Although this underlines the complexity of understanding the immune mechanisms related to pathogenicity or protection induced by mycobacteria, we clearly found distinct transcript and protein secretion profiles in human macrophages infected with rBCG-LTAK63 compared to cells infected with either BCG or *Mtb*. Previously, rBCG-LTAK63 vaccination was shown to increase the recruitment of neutrophils, macrophages, and lymphocytes and induce enhanced innate immune response with higher levels of mediators of macrophage activation (nitric oxide, hydrogen peroxide, and inflammatory cytokines) in vivo in a murine TB infection model [16]. Here, our data in human in vitro infection models indicate an important immunological profile induced in human macrophages by rBCG-LTAK63 (Figure 5) that can be correlated with a protective immune response against TB. Further studies will be needed to confirm this hypothesis.

The distinct immunological profiles induced in human macrophages indicate that the mechanism of protection may be different from BCG and the currently studied vaccines, reinforcing the potential of rBCG-LTAK63 as a vaccine candidate.

## 5. Patents

I.P.N. and L.C.C.L. have a patent application involving rBCG-LTKA63 use as *Mtb* vaccine.

## Data Availability

The data presented in this study are available on request from the corresponding author.

## Supplementary Materials

The following supporting information can be downloaded at: https://www.mdpi.com/article/10.3390/vaccines10060831/s1, Figure S1: Kinetics of the intracellular bacilli survival and rBCG-LTAK63 stability in M1 and M2 macrophages infection; Figure S2: Genes analyzed by dcRT-MLPA and their predominant functions; Table S1: Statistical analysis of expression of all genes evaluated by dcRT-MLPA in M1 macrophages in response to infection with rBCG-LTAK63, BCG, or *Mtb* H37Rv compared to uninfected macrophages; Table S2: Statistical analysis of expression of all genes evaluated by dcRT-MLPA in M2 macrophages in response to infection with rBCG-LTAK63, BCG, or *Mtb* H37Rv compared to uninfected macrophages.
