# Supplementary material for: Recombinant BCG-LTAK63 Vaccine Candidate for Tuberculosis Induces an Inflammatory Profile in Human Macrophages
Source: Vaccines (Basel). 2022 May 24;10(6):831. doi: 10.3390/vaccines10060831 (PMC9227035; doi:10.3390/vaccines10060831)
Supplement: Supplementary file 1 [file vaccines-10-00831-s001.zip › Supplementary Figures.pdf]

Supplementary Figures

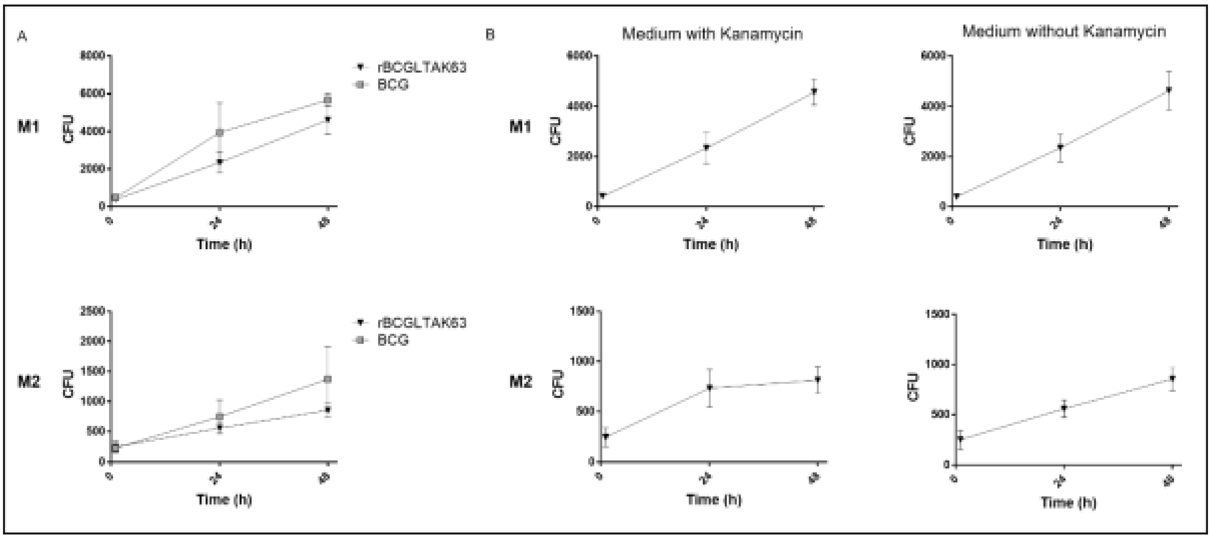

Supplementary Figure S1. Kinetics of the intracellular bacilli survival and rBCG-LTAK63 stability in M1 and M2 macrophages infection. M1 and M2 macrophages from 4-7 different donors were infected with rBCG-LTAK63 or BCG (MOI 10). (A) The intracellular bacteria were recovered 1 h, 24 h or 48 h after infection. (B) The bacteria recovered from the macrophages infected with rBCG-LTAK63 was plated in 7H10 with kanamycin or without kanamycin.

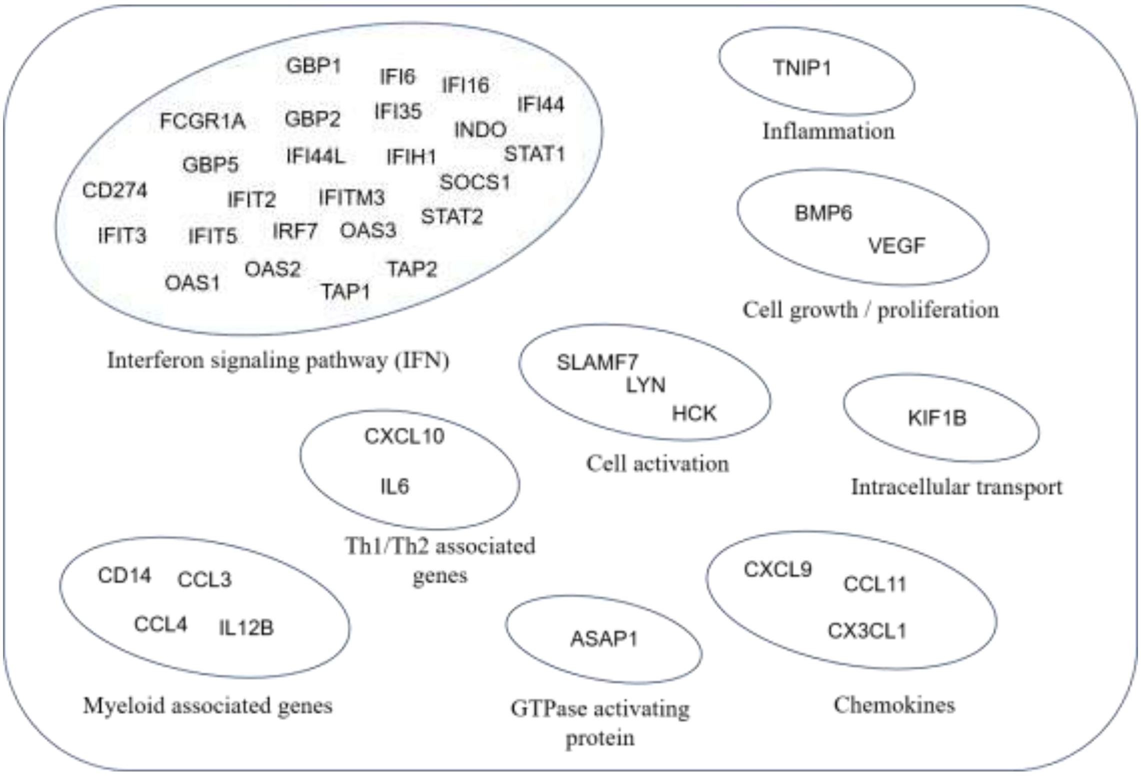

Supplementary Figure S2. Genes analyzed by dcRT-MLPA and their predominant functions.
